# Evaluation of the effect of neoadjuvant chemotherapy on the planned resectability of extremity soft-tissue sarcomas

**DOI:** 10.1302/2633-1462.65.BJO-2025-0026.R1

**Published:** 2025-05-16

**Authors:** Simon Tournemine, Sylvie Bonvalot, Jean-Yves Mary, Dimosthenis Andreou, David Biau

**Affiliations:** 1 Université Paris-Cité, INSERM U1153, AP-HP, Hôpital Cochin, Service de Chirurgie Orthopédique, Paris, France; 2 Department of Surgical Oncology, Institut Curie, Paris, France; 3 INSERM U1342, Team ECSTRRA, Université Paris Cité, Hôpital Saint-Louis, Paris, France; 4 Department of Orthopaedics and Trauma, Institute for Interdisciplinary Sarcoma Treatment and Research, Department of Orthopedic Oncology and Sarcoma Surgery, University Hospital Essen, Essen, Germany

**Keywords:** Soft-tissue sarcoma, Imaging, Chemotherapy, Surgical technique, Neoadjuvant chemotherapy, extremity soft-tissue sarcomas, chemotherapy, Soft-tissue sarcomas, amputations, anatomical structures, surgical resection, femoral artery, sciatic nerve, femur

## Abstract

**Aims:**

In this study, we explore whether neoadjuvant chemotherapy influences the surgical resection strategy devised by surgeons for high-grade soft-tissue sarcoma.

**Methods:**

A total of 12 experienced soft-tissue sarcoma surgeons rated patients who underwent neoadjuvant chemotherapy for a soft-tissue sarcoma of the thigh. Cases were randomly assigned to surgeons, such that each surgeon rated three out of the 12 cases, and each case was rated by three out of 12 surgeons (n = 36 ratings before and after chemotherapy). Surgeons were asked which surgical technique they would use: amputation; and if not, resection or dissection of critical anatomical structures in close proximity to the tumour (sciatic nerve, femoral artery, and femur). Pre- and post-chemotherapy ratings were then compared to test if chemotherapy changed the surgery aggressiveness anticipated by the surgeons.

**Results:**

Tumour volume increased in 9/12 cases (75%). Ratings as amputation were discordant in 5/36 cases (14%) before and after chemotherapy. The surgical technique planned by surgeons before and after chemotherapy regarding critical anatomical structures were discordant in five (14%), eight (22%), and six of 36 ratings (17%) for the sciatic nerve, the femoral artery, and the femur, respectively. Overall, a similarly aggressive surgery was planned by surgeons in nine, six, and eight cases for the sciatic nerve, the femoral artery, and the femur, respectively, which is significantly more than that expected due to chance alone. A more aggressive surgery was anticipated in five of 36 cases (14%).

**Conclusion:**

Despite tumour growth being observed in 75% of cases, the surgical resection strategy devised after neoadjuvant chemotherapy remained notably similar to the one devised prior to neoadjuvant chemotherapy for critical anatomical structures. However, ‘switchers’, namely patients identified as being at risk of needing an amputation if the tumour experiences slight growth, should undergo conservative surgery initially, followed by chemotherapy.

Cite this article: *Bone Jt Open* 2025;6(5):553–559.

## Introduction

Soft-tissue sarcomas encompass a rare group of tumours of over 100 different types of malignant tumours originating from mesenchymal cells, impacting approximately 13,400 individuals annually in the USA.^1^ It accounts for less than 1% of all cancers.^2^ Soft-tissue sarcomas may be diagnosed at any age and affect any part of the body, with some predominance in the lower limb. The five-year overall survival (OS) rate stands at approximately 55%, with histology, grade, and size emerging as the most significant prognostic factors.^3^

To date, the mainstay of treatment for a localized soft-tissue sarcoma is surgery. Radiotherapy (RT) is part of the standard of care in high-risk extremity soft-tissue sarcoma (ESTS), which has been validated by two randomized trials and multiple cohort studies.^4-6^ The use of chemotherapy remains, however, a subject of debate since the seminal large individual patient meta-analysis of adjuvant doxorubicin-based chemotherapy failed to show a significant benefit on patient OS.^7^ Recently, re-evaluation of the results from the EORTC-STBSG 62931 randomized trial showed that patients with a predicted OS ≤ 51% had better outcomes when treated with adjuvant chemotherapy.^8^ Similarly, a re-analysis of data from the ISG-STS 1001 randomized study demonstrated a potential absolute OS gain of nearly 10% with neoadjuvant chemotherapy in patients with a Sarculator-predicted OS ≤ 60%.^9^ Therefore, currently, the global strategy has pivoted towards adopting neoadjuvant chemotherapy in selected high-risk patients.^8-10^

At the imaging level, chemotherapy is rarely associated with significant volumetric changes. Indeed, in the recent ISG-STS 1001 trial, no complete responses were observed. Of the 230 patients evaluable for response, 10% had a partial response, 80% had stable disease, and 10% had progressive disease according to Response Evaluation Criteria in Solid Tumors (RECIST).^11^ At the histological level, it appears that neoadjuvant chemotherapy contributes to the development of a pseudocapsule around the sarcoma and decreases the probability of finding malignant cells within and beyond the pseudocapsule.^12,13^ Eventually, when addressing the complexity of tumour removal, surgeons rely on preoperative MRI assessments. They examine various factors including the tumour’s size, surrounding anatomical barriers, critical structures such as bones, vessels, and nerves, as well as any soft-tissue oedema present. Surgeons will assess whether the tumour is suitable for a conservative surgical resection. If deemed appropriate, they will then select the necessary surgical techniques for the procedure.^14-16^ In 5% of cases or fewer, they may choose amputation to ensure compliance with the oncological principle of achieving an R0 resection.^17-19^ Hence, if neoadjuvant chemotherapy alters tumour volume and its relationship with surrounding structures, it can also impact the planning of surgical resection.

In this study, we explored the impact of neoadjuvant chemotherapy on MRI-based surgical planning for soft-tissue sarcoma. Our aim was to determine whether neoadjuvant chemotherapy influences the surgical resection strategy devised by surgeons for high-grade soft-tissue sarcoma located in the thigh.

## Methods

### Study design

Selection of cases: In total, 12 cases were selected from a soft-tissue sarcoma database (SATIMO; Assistance Publique - Hôpitaux de Paris, France) of more than 450 patients by two of the authors (ST, DB). To obtain a homogenous subset of patients, we (ST, DA, SB, DB) decided to include patients who presented with a high-grade, deeply seated soft-tissue sarcoma of the thigh, who underwent neoadjuvant chemotherapy and completed at least two cycles, did not receive radiotherapy in the meantime, who had adequate MRI studies before and after neoadjuvant chemotherapy (see below), and for whom a conservative surgery was intended based on the discussion between practitioners at the multidisciplinary tumour meeting. Myxoid liposarcoma and atypical lipomatous tumours were excluded. Cases were selected in chronological order until 12 cases with adequate imaging studies were obtained. Patients consented to share their data, and the study was conducted according to the ethical principles set forth in the 1964 Declaration of Helsinki^20^ and the French reference methodology MR-004 to the CNIL (Commission Nationale Des Libertés Informatiques; no. 2235583).

MRI studies: All cases should have undergone an MRI before the start of chemotherapy (within two weeks) and after the end of chemotherapy (within two weeks). The pre- and postoperative MRIs included axial T1-weigthed non fat-sat, T2-weighted (or water-sensitive equivalent sequences) fat-sat, a T1-weighted fat-sat gadolinium-enhanced sequence, and last T1-weigthed coronal or sagittal sequences. Dates and names were removed from the images, and no indication of whether the MRI study was performed before or after the neoadjuvant chemotherapy was available. Tumour volume was measured before and after chemotherapy by multiplying its dimensions along the three standard orthogonal axes.

Selection of surgeons: A total of 12 soft-tissue sarcoma surgeons were selected on a voluntary basis among the surgeons of the European MusculoSkeletal Oncology Society and French Sarcoma Group (see Acknowledgements). All surgeons were consultants with many years of experience in sarcoma surgeries, and all agreed to participate.

**Fig. 1:**
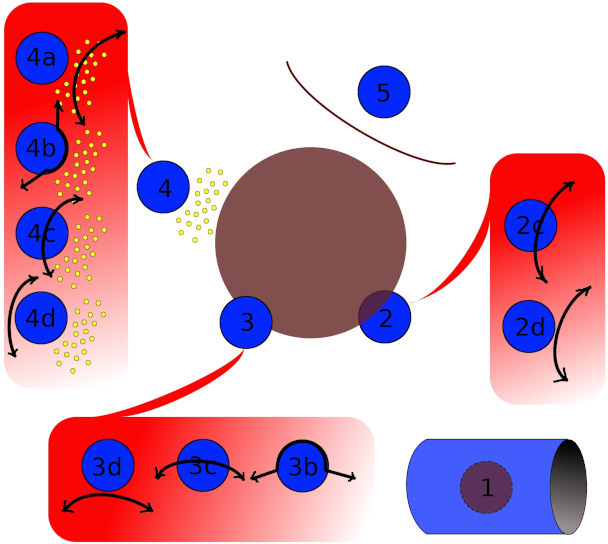
Schematic representation of surgical dissection types. Brown circle: tumour. Blue circle: anatomical structure of interest. Arrows: path of surgical dissection. a: extra-fascial dissection; b: sub-fascial dissection; c: partial resection; d: complete resection. Case 1: the tumour is embedded inside the anatomical structure (for example, a sub-cutaneous sarcoma); case 2: the tumour invades the anatomical structure (for example, bone invasion of a soft-tissue sarcoma); case 3: the tumour abuts the structure; case 4: there is a fatty plane between the tumour and the anatomical structure; and case 5: the tumour is separated from the anatomical structure by another structure.

Ratings of cases by surgeons: To avoid the readings of all cases by all surgeons, case readings were randomly assigned to surgeons such that each surgeon rated three out of the 12 cases, and each case was rated by three out of the 12 surgeons, leading to a total of 36 ratings. Surgeons were asked first whether they chose limb salvage or amputation (transfemoral amputation (TA), or hip disarticulation (HD)); then, if they planned on using radiation (data not shown) if the resulting margins were as anticipated (pre- or postoperative, regardless); finally, in case of limb salvage, they were asked to detail how they would deal with critical anatomical structures in close proximity to the tumour (sciatic nerve, femoral artery, and femur). Four dissection types were possible (Figure 1): extra-fascial dissection (ED) if the dissection plane was between the tumour and the covering fascia of the structure; sub-fascial dissection (SD) if the dissection plane was just under the fascia (periosteum, adventicia, epineurium, epimysium) with the corresponding surgical techniques: subperiosteal dissection,^15^ subadventicial dissection,^16^ subepineural dissection,^14^ and subepimysial dissection; partial resection (PR) when the dissection plane was inside the structure (for instance, if performing a geometrical osteotomy of the bone to resect the tumour, or when performing a partial arterial resection); and a complete resection (CR) if none of the structures could be spared (for instance, when removing the sciatic nerve for a posterior compartment tumour or the superficial femoral artery for a tumour of the popliteal fossa). When structures were far away from the tumour (not relevant on the rating form), the dissection was considered as the easiest, namely extra-fascial. The rating form with detailed explanations was conceived by ST, DA, SB, DB, and sent to surgeons to collect their anticipated surgery for each rated case (Supplementary Material).

### Statistical analysis

Tumour volume was expressed as median (IQR) and compared before and after chemotherapy using the Mann-Whitney U test test.^21^ Tumour response according to RECIST, and the number and proportion of cases showing an increase or decrease in tumour volume, was also reported.^11^

For simplicity, the proportion of each anticipated surgery was expressed as the number of ratings anticipating this surgery divided by the total number of ratings, namely 36, ignoring that each case was not rated by all surgeons.

To account for the correlation between cases and between raters, the ratings of one case by its three raters was summarized by a series of three numbers: the number of raters who anticipated a less aggressive surgery, the number of raters who anticipated a similarly aggressive surgery, and the number of raters who anticipated a more aggressive surgery, compared to the surgery anticipated before chemotherapy. Each number varies between 0 and 3 and their sum is equal to 3. As an example, for one case, the series 1-2-0 means that one surgeon anticipated a less aggressive surgery, two surgeons anticipated a similarly aggressive surgery, and none of the three surgeons anticipated a more aggressive surgery after chemotherapy; the series 0-3-0 means that all three surgeons anticipated a similarly aggressive surgery after chemotherapy than before chemotherapy. An anticipated surgery was considered less aggressive if it was downgraded from amputation (HD or TA) to either resection (CR or PR) or dissection (SD or ED), or from resection (CR or PR) to dissection (SD or ED). A surgery was considered more aggressive if it was upgraded from dissection (SD or ED) to either resection (CR or PR) or amputation (HD or TA), and from resection (CR or PR) to amputation (HD or TA). The anticipated surgery was considered similar when it did not change after chemotherapy. All ten possible series that can be observed are described in Table I. If chemotherapy had no effect on the anticipated surgeries, the null hypothesis, then all ten series had an equal likelihood of occurrence and therefore the probability of observing any series was 1/10 = 0.1. One series was observed for each out of the 12 cases. To test if chemotherapy changed the surgery aggressiveness anticipated by the surgeons, we calculated from the binomial distribution with n = 12, p = 0.1 the probability of occurrence of as many 0-3-0 or more than the number of observed 0-3-0, assuming the null hypothesis, i.e. no change of the surgery aggressiveness anticipated by the surgeons. The corresponding p-values were computed from the standard normal distribution table. According to the very low number of series with discordant surgery aggressiveness anticipated by the surgeons before and after chemotherapy, no p-value was calculated to compare these situations. Moreover, for each of the 12 cases, a more aggressive surgery was defined when a decision for amputation was considered after chemotherapy when it was not before surgery; and when a conservative surgery was opted for, if the decision for a more aggressive surgery in at least one of the three structures was decided after chemotherapy.

**Table I.** Series that can be observed for each anatomical structure according to the ratings of all three raters.

Surgeon 1	Surgeon 2	Surgeon 3	Series notation
Less aggressive	Less aggressive	Less aggressive	3-0-0
Less aggressive	Less aggressive	Similarly aggressive	2-1-0
Less aggressive	Less aggressive	More aggressive	2-0-1
Less aggressive	Similarly aggressive	Similarly aggressive	1-2-0
Less aggressive	More aggressive	More aggressive	1-0-2
Less aggressive	Similarly aggressive	More aggressive	1-1-1
Similarly aggressive	Similarly aggressive	Similarly aggressive	0-3-0
Similarly aggressive	Similarly aggressive	More aggressive	0-2-1
Similarly aggressive	More aggressive	More aggressive	0-1-2
More aggressive	More aggressive	More aggressive	0-0-3

## Results

**Fig. 2:**
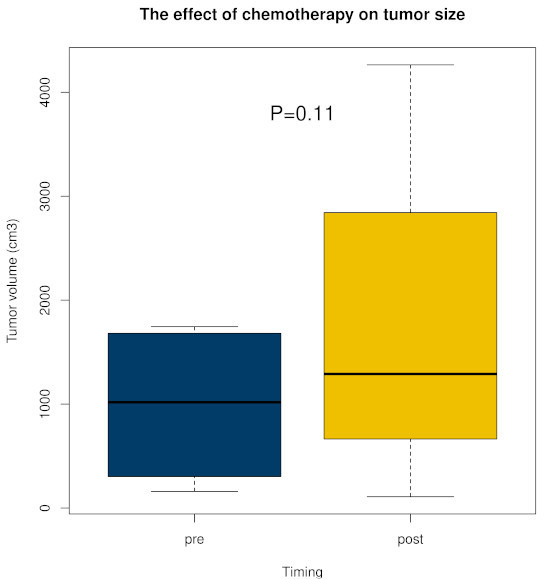
The effect of chemotherapy on tumour volume. Box and whiskers plot representing the minimum, first quartile, median, third quartile, and maximum value.

Tumour volume difference showed a median of 174 cm^3^ (IQR -38 to 716, p = 0.109; Figure 2). Among the 12 cases, tumour volume increased in nine cases (75%) and decreased in three cases (25%). According to RECIST, seven cases were rated as stable disease and five as progressive disease. Prior to chemotherapy, out of 36 ratings, two (6%) indicated a preference for amputation or disarticulation, each associated with different cases and raters. Post chemotherapy, three ratings (8%) favoured amputation or disarticulation, corresponding to two different cases and two distinct raters; notably, only one of these cases had been rated for amputation or disarticulation preoperatively.

**Fig. 3:**
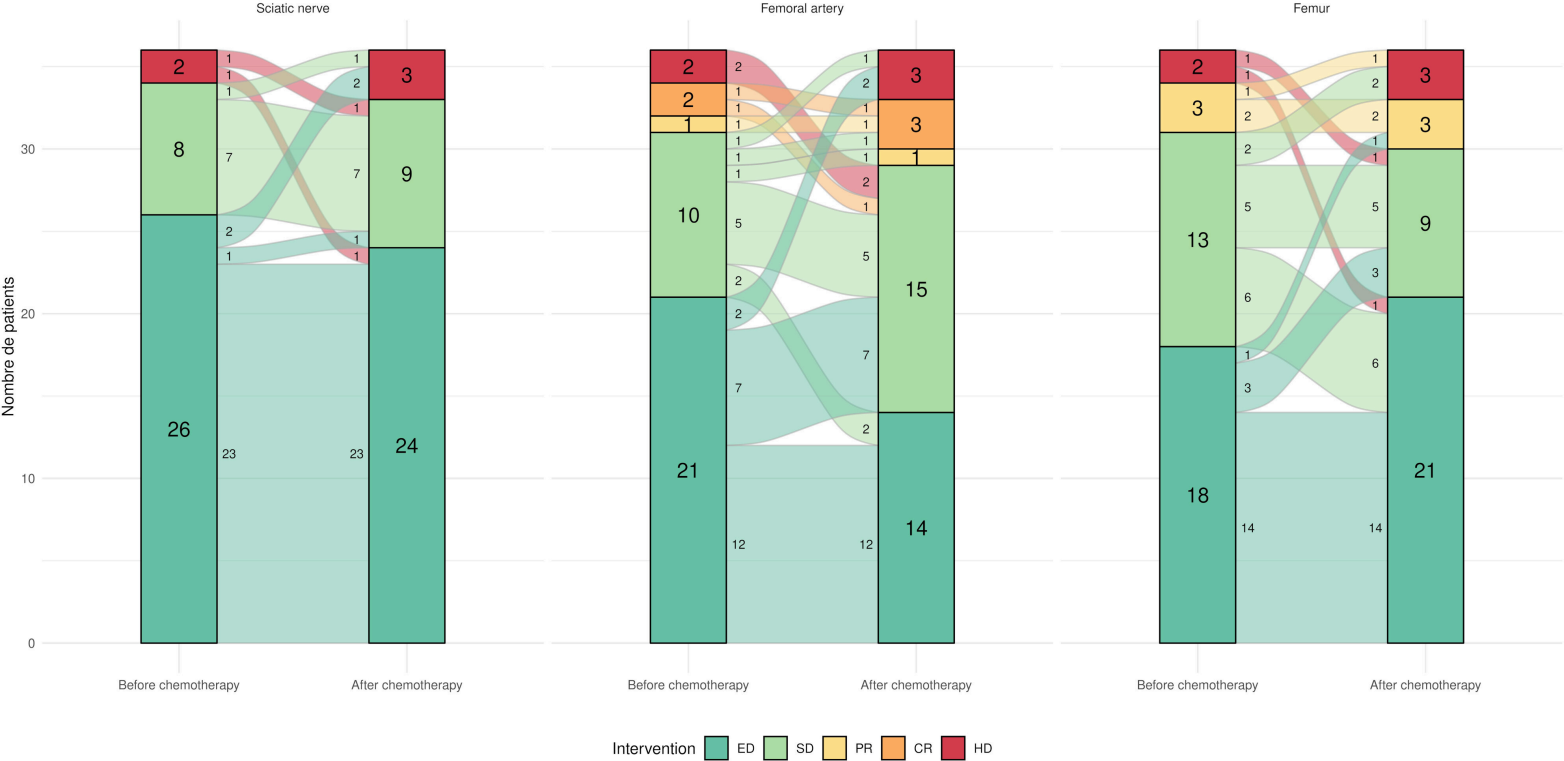
Changes in the surgical techniques. Changes in the surgical techniques chosen by the 12 readers for the 12 cases, before and after chemotherapy, for the femur, the sciatic nerve, and femoral artery (Sankey diagram). CR, complete resection; ED, extra-fascial dissection; HD, hip disarticulation; PR, partial resection; SD, sub-fascial dissection.

Concerning the sciatic nerve, prior to chemotherapy, 34 ratings were in favour of extra-fascial/sub-fascial dissection, and two (6%) in favour of an amputation/disarticulation (see above). Post chemotherapy, 33 ratings (92%) were in favour of extra-fascial/sub-fascial dissection, and three (8%) in favour of an amputation/disarticulation (see above). Of the 36 ratings, five (14%) were discordant between the pre and postchemotherapy (Figure 3a). Overall, we observed nine times the combination 0-3-0, indicating a similarly aggressive surgery by all three raters (p < 0.001).

Concerning the femoral artery, prior to chemotherapy, 31 ratings (86%) were in favour of extra-fascial/sub-fascial dissection, three (8%) were in favour of a partial/complete resection, and two (6%) in favour of an amputation/disarticulation (see above). Post chemotherapy, 29 ratings (81%) were in favour of extra-fascial/sub-fascial dissection, four (11%) were in favour of a partial/complete resection, and three (8%) in favour of an amputation/disarticulation (see above). Of the 36 ratings, eight (22%) were discordant between pre- and post chemotherapy (Figure 3b). Overall, we observed six times the combination 0-3-0, indicating a similarly aggressive surgery by all three raters (p < 0.001).

Concerning the femur, prior to chemotherapy, 31 ratings (86%) favoured extra-fascial/sub-fascial dissection, three (8%) were in favour of a partial/complete resection, and two (6%) in favour of an amputation/disarticulation (see above). Post chemotherapy, 30 ratings (83%) were in favour of extra-fascial/sub-fascial dissection, three (8%) were in favour of a partial/complete resection, and three (8%) in favour of an amputation/disarticulation (see above). Of the 36 ratings, six (17%) were discordant between the pre and postchemotherapy (Figure 3c). Overall, we observed eight times the combination 0-3-0, indicating a similarly aggressive surgery by all three raters (p < 0.001). Overall, a more aggressive surgery was decided in 13.9% of cases (five of 36).

## Discussion

While surgical resection remains the primary treatment approach for localized soft-tissue sarcoma, neoadjuvant chemotherapy is an option in select cases.^11^ Considering the impact of neoadjuvant chemotherapy on soft-tissue sarcoma,^12,13,22^ we investigated whether it influences surgical planning. Overall, our findings suggest that neoadjuvant chemotherapy was significantly associated with maintaining the status quo in resection planning. Surgeons generally maintained similar resection plans after neoadjuvant chemotherapy compared to those made prior to treatment. A more aggressive surgery was anticipated in less than 14% of cases.

Indeed, there are few reasons to upgrade a surgery, as the majority of soft-tissue sarcomas typically do not invade, but rather push and compress, surrounding healthy tissues as they grow, forming a pseudocapsule. Consequently, critical structures are often conducive to surgical dissection and salvage, unless the tumour completely encases the structure to the extent that access for the surgeon becomes unfeasible. However, prior to that point, surgeons deemed it feasible to excise the tumour via extra-fascial or sub-fascial dissection in over 85% of cases. It has been shown that, even for tumours pushing against a structure, with appropriate sub-fascial dissection^14-16^ and adjuvant radiotherapy, oncological outcomes were acceptable.^23^ In the present study tumour volume increased from a median 1,017 cm^3^ (IQR 346 to 1,650) prior to chemotherapy to a median 1,290 cm^3^ (IQR 708 to 2,828) post chemotherapy; according to RECIST, seven cases were rated as stable disease and five as progressive disease. This is in accordance with the recent randomized, open-label, phase III trial comparing the administration of histology-tailored (HT) neoadjuvant chemotherapy to the administration of standard anthracycline plus ifosfamide (A + I) neoadjuvant chemotherapy in high-risk soft-tissue sarcoma (STS) of a limb or the trunk wall.^11^ Of the 230 patients, 23 (10%) had a partial response (16 of 117 patients (13.6%) in the A + I arm; seven of 113 patients (6.2%) in the HT arm, whereas 184 of the 230 patients (80%) had stable disease (93 of 117 patients (79.5%) in the A + I arm; 91 of 113 patients (80.5%) in the HT arm). Of 230 patients, 23 (10%) had progressive disease (eight of 117 patients (6.8%) in the A + I arm; 15 of 113 patients (13.3%) in the HT arm).

Amputation or disarticulation is considered a last-resort procedure.^24^ In our series, surgeons would have performed an amputation or disarticulation in a little less than 10% of cases (6% before chemotherapy and 8% after), which is in keeping with figures reported in the literature. Ghert et al,^18^ in a retrospective series of 413 patients operated on for a soft-tissue sarcoma, performed 25 (6%) amputations. Similarly, Smith et al^19^ reported on a series of 69 patients (4.1%) undergoing an amputation for soft-tissue sarcoma. To the best of our knowledge, only Kirilova et al^25^ reported the detailed reasons for performing an amputation in a retrospective series of 120 cases: multiple compartments involved (56%), size (27%), neurovascular involvement (9%), bone involvement (6%), and combined reasons (3%) were declared as the reason for amputation. The comparatively, slightly higher rate of amputation or disarticulation observed in our series may be attributed to patient selection. All patients underwent chemotherapy for high-grade tumours, which are more likely to be larger and more aggressive than the average.

Some patients will switch during neoadjuvant chemotherapy from a conservative to a non-conservative surgery. This transition occurred in five out of 36 ratings, constituting 14% of cases. In two cases, the surgeon downgraded the planned surgery to a conservative approach, while in three cases, the surgeon upgraded the planned surgery to an amputation or disarticulation from a conservative approach. Particular attention should be payed during multidisciplinary meetings to identify these potential switchers. Patients for whom the tumour is at a tipping point, where even a slight increase in size could lead to an amputation, should be prioritized for surgical intervention. In such situations, postoperative chemotherapy can be considered if deemed necessary. Conversely, patients for whom a conservative surgery is not feasible due to local tumour invasion may undergo chemotherapy as the initial treatment approach. Subsequent decisions regarding surgery can then be determined based on the evolution of the tumour.

This study has several limitations. First, the surgeons provided their judgement based on images alone, rather than based on intraoperative findings. Although oncological surgeries are performed according to a preoperative plan with minor adjustments during the resection, a difference between what is planned and what is performed sometimes occurs. These differences would likely affect both pre- and postoperative ratings similarly, so the comparison still stands. Second, we limited our sample to high-grade sarcomas of the thigh, and our findings may not apply to other anatomical areas. However, we believe that surgical considerations, such as how to manage tumours in proximity to nerves or bones, remain relatively similar in the appendicular skeleton. Third, raters were blinded to the timing of chemotherapy in order to limit bias. However, chemotherapy is known to have some effect at the histological level with less probability of finding tumour cells within and beyond the pseudocapsule.^12,13^ Therefore, surgeons could act differently on a morphologically similar tumour (in volume and in relation to critical structures) if they knew whether the patient had received chemotherapy or not. Finally, preoperative radiotherapy is regularly used as an adjuvant to surgical resection, and a similar study should be performed including patients undergoing neoadjuvant radiation.

In conclusion, the surgical resection strategy formulated by surgeons for high-grade soft-tissue sarcoma located in the thigh after neoadjuvant chemotherapy remained notably similar to the strategy devised prior to neoadjuvant chemotherapy.

Following neoadjuvant chemotherapy, surgeries were upgraded in less than 14% of cases, despite a significant trend of tumour growth observed during chemotherapy and affecting 75% of cases. This underscores the importance of clinical and radiological monitoring of patients undergoing chemotherapy to promptly switch to surgery in the event of significant progression.

Indeed, surgeons should exercise caution with‘switchers’, referring to patients whose tumours are at a critical point between conservative surgery and amputation. Patients identified as switchers should undergo conservative surgery initially, followed by chemotherapy as deemed necessary.

**Take home message**

- Neoadjuvant chemotherapy for high-grade soft-tissue sarcoma is significantly associated with maintaining the status quo in resection planning.

- Patients identified as being at risk of needing an amputation if the tumour experiences slight growth ('switchers') should undergo conservative surgery initially.

## Data Availability

The data that support the findings for this study are available to otherresearchers from the corresponding author upon reasonable request.
